# Effects of Ca Sprays on Fruit Ca Content and Yield of Tomato Variety Susceptible to Blossom-End Rot

**DOI:** 10.3390/plants12081640

**Published:** 2023-04-13

**Authors:** Andis Karlsons, Anita Osvalde, Gunta Cekstere, Laura Āboliņa

**Affiliations:** Institute of Biology, University of Latvia, LV-1004 Riga, Latvia

**Keywords:** artificial lighting, autumn-spring season, calcium, hydroponics, plant nutrient status, *Solanum lycopersicum* L., variety ‘Beorange’

## Abstract

Several factors are involved in the incidence of blossom-end rot (BER) in tomato fruit, but the main one is insufficient Ca uptake and transport through the plant, resulting in Ca deficiency in the fruit. Sprays of Ca-containing products are considered to be a possible measure to overcome the local Ca deficiency in tomato fruit. Therefore, the main objective was to evaluate the effectiveness of additional Ca supply to tomato fruits for increasing Ca content and reducing fruit damage. Sprays of five different commercial preparations containing (Brexil Duo, Calmax Zero N, Ca(NO_3_)_2_, CaCl_2_) or promoting (Greenstim) Ca uptake were tested using BER-sensitive large-fruit variety ‘Beorange’. The experiment was conducted in the commercial greenhouse ‘Getlini EKO’, Latvia, during the autumn–spring season of 2020/2021 under controlled conditions, eliminating the adverse impact of external factors. The results revealed that none of the preparations were effective in increasing Ca content, preventing BER, and did not promote the tomato yield. As good agricultural practices were followed in the greenhouse to manage BER, we concluded that a non-marketable yield of around 15% should be expected for ‘Beorange’ when grown under artificial light, possibly due to the impact of abiotic stresses and genetically determined susceptibility.

## 1. Introduction

Fruit blossom-end rot (BER) is a common and serious problem in vegetable production worldwide. In particular, this physiological disorder can lead to a drastic loss in the yield and quality of tomato fruits (*Solanum lycopersicum* L.) [1,2]. BER generally appears as a visible black necrotic spot at the blossom-end of the fruit. Several factors as environmental conditions, nutrient antagonism, stress-induced production of reactive oxygen species (ROS), the balance of plant phytohormones, and genetic variability are discussed regarding the incidence of BER [1,3,4]. In addition, all these factors are involved in the inhibition of Ca accumulation or abnormal regulation of cellular Ca partitioning in plants [5]. Therefore, the main factor considered in BER development is the insufficient uptake and transport of Ca through the plant, which results in Ca deficiency in the fruit tissue, especially in the distal proportion of the fruit [6]. Possible Ca deficiency in the plant is largely determined by low Ca availability from the growing medium, low root activity, and transpiration rate [7]. Research on greenhouse tomato production has demonstrated that unfavourable growth conditions such as drought, irregular water conditions, salinity, high light intensity and temperature, and nutrient imbalance also can induce BER. These abiotic stresses can not only cause oxidative stress but also inhibit Ca uptake by plants, thereby contributing to the development of Ca-deficient damage in fruit [3,8,9]. Increased concentrations of nutrients that antagonise Ca uptake can also increase the risk of physiological Ca disorders. In this respect, excessive K and Mg fertilisation has been found to increase the incidence of BER in tomatoes [1,5,6]. As Ca plays an important role in the stabilisation of cell wall pectin and plasma membranes [7], typical symptoms of Ca-deficiency are the disintegration of cell walls and the disruption of affected tissues characteristic for BER-affected fruits.

A set of factors are important in the choice of varieties for tomato production, especially in the greenhouse conditions under the autumn–spring growing cycle. In addition to high productivity and compliance with consumer requirements in terms of fruit size, colour, and taste, resistance to BER can be crucial [10,11]. Although tomato varieties generally differ in their sensitivity to BER, large-fruited and elongated varieties are reported to have significantly higher BER levels than rounded, flattened, medium- and small-sized ones [4,12]. These differences can be explained by the rapid expansion of young fruits and higher growth rates in large-fruited varieties, thus diluting the Ca concentration in the fruit distal part and increasing the sensitivity to BER. Although the cultivation of large-fruited tomatoes is more complex and non-marketable yields can reach a significant proportion of the harvest, this type of tomato is very popular with consumers and has a high market price, which makes it profitable. Large fruit size is a particularly important trait for fresh-market tomato production [13,14].

Studies on tomato growing in greenhouses have shown that an insufficient Ca level in the growing medium/nutrient solution is unlikely and rarely causes BER. Physiological Ca deficiency in plants with a sufficient supply of Ca to the roots is more often determined by environmental and genetic factors, as well as nutrient imbalance [2,3,5,15]. Therefore, it is important to find effective ways to increase the Ca content in fruits, especially for BER susceptible varieties. In this regard, the additional use of Ca-containing products is considered to be a possible measure to overcome the local Ca deficiency in tomato fruit during the period of rapid cell expansion. Although studies have been performed on Ca sprays on fruits to control BER [16,17,18], very little information is available on the effective types and doses of Ca sources. There are several commercial products supplying calcium to plants, but there is insufficient evidence to recommend their use in the management of BER. Most reports on positive results for greenhouse tomatoes are based only on sprays of CaCl_2_ solution as a part of management practice [2,18,19,20].

Therefore, the main objective of this study was to evaluate the effectiveness of additional Ca supply to tomato fruit as a means of increasing Ca content and reducing fruit damage caused by BER. To achieve this goal, sprays of five different preparations containing Ca (Brexil Duo, Calmax Zero N, Ca(NO_3_)_2_, CaCl_2_) or promoting Ca uptake (Greenstim), on tomato fruits were tested using BER-sensitive large-fruit size variety ‘Beorange’.

The ‘Beorange’ variety is the only large-fruited orange tomato variety that is grown hydroponically in the autumn–spring season in Latvia. It is popular with consumers and its cultivation is economically beneficial. Therefore, this study was conducted not only to gain scientific knowledge but also to provide practical guidance to vegetable growers.

## 2. Materials and Methods 

### 2.1. Study Site and Plant Growth Conditions

The experiment was conducted in a commercial greenhouse Ltd. ‘Getlini EKO’, located near Riga, Latvia (hemiboreal climatic zone), during the autumn–spring season of 2020/2021, using solar plus supplemental high–pressure sodium lighting. In this greenhouse, the plants were provided with appropriate and controlled conditions, eliminating the adverse effects of external factors. Orange large fruit-sized tomato variety ‘Beorange F1’ (average fruit weight 180–200 g) was hydroponically grown on rockwool substrate (Grodan, Netherland). The potential incidence of BER for the ‘Beorange’ variety has been reported to be around 13–15% [21,22]. Tomato seedlings were transplanted into slabs in mid-August with a density of 2.5 plants per m^−2^. Bumblebees (*Bombus terrestris* L.) were used for pollination. 

**Figure 1:**
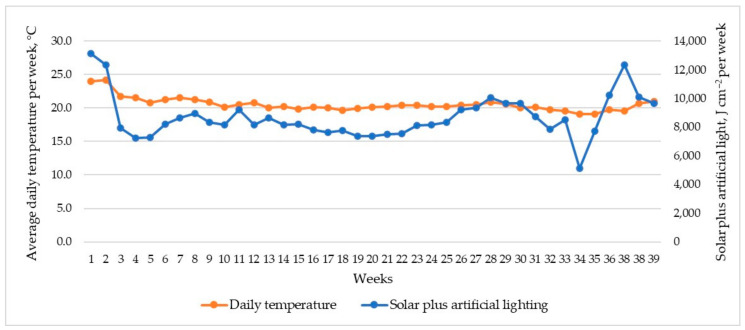
Average daily temperatures and solar plus artificial lighting per week during ‘Beorange’ tomato production cycle from August 2020 to May 2021.

All agrotechnical measures were carried out following current recommendations for soilless tomato cultivation [23]. During the tomato crop cycle (August 2020–May 2021), the average day/night temperature was 22.0/18.8 °C, respectively. The intensity of solar plus artificial light ranged from 730 to 1880 J cm^−2^ day^−1^, with an average value of 1240 J cm^−2^ day^−1^. The average daily exposure time to artificial lighting in the October–March period was 16.5 h. Average daily temperatures and solar plus artificial lighting per week during ‘Beorange’ tomato production cycle from August 2020 to May 2021 are shown in Figure 1. The autumn–spring growing season was generally not characterised by short periods of high light intensity and temperature, which would lead to very rapid fruit growth. 

Nutrient solution of the following chemical composition (mg L^−1^): <20 N–NH_4_, 230 N–NO_3_, 40 P, 395–495 K, 275 Ca, 60 Mg, 120–180 S–SO_4_, 1.98 Fe, 0.32 Mn, 0.75 Zn, 0.13 Cu, 0.05 Mo, 0.45 B was used. The pH was adjusted to 5.3–5.5 and the EC values were maintained at an average level of 3.3 mS cm^−1^. To optimise Ca uptake by roots, high salt concentrations (>5 mS cm^−1^), excessive ammonium N form (>10% of total N) in the nutrient solution, and excessive dryness in the root zone were prevented.

Leaf thinning was performed for all treatments at the end of the growing cycle to reduce the leaf–fruit ratio. Defoliation was started at the end of January when there was an increasing tendency to the proportion of fruits damaged by BER.

### 2.2. Ca Preparations and Plant Treatments

To test the possibilities to increase Ca content in tomato fruits, treatments with 5 different commercially available preparations containing Ca or promoting Ca uptake were applied. The application rates were chosen according to the manufacturer’s instructions. The spraying treatments were as follows:

1. Control—deionized water;
2. An aqueous 0.2% solution of Brexil Duo (Ca 12.9%, Mg 2.4%, B 0.5%, Cu 0.5%, Mn 2%, Zn 2%; water-soluble foliar fertiliser, nutrients complexed with lignin sulphonate, Valagro SDS, Atessa, Italy);
3. An aqueous 0.2% solution of Greenstim (Ca 0%, a preparation promoting Ca uptake, 97% glycine betaine; Verdera, Espoo, Finland);
4. An aqueous 0.5% solution of Calmax zero N (Ca 7.13%, B 0.33%, Cu 0.04%, Fe 0.05%, Mn 0.1%, Mo 0.001%, Zn 0.02%; concentrated suspension containing CaO, micronutrients chelated with EDTA; Omex Agrifluids Ltd., King’s Lynn, UK);
5. An aqueous 0.5% solution of Ca(NO_3_)_2_ (Ca 19%; Yara Latvia Ltd., Riga, Latvia);
6. An aqueous 0.5% solution of CaCl_2_ (Ca 28%; Yara Latvia Ltd., Riga, Latvia).

From the beginning of October to the end of March, solutions were manually sprayed directly on the upper trusses with young fruits (immediately after the fruit initiations, before symptoms of BER) once a week. Therefore, all upper trusses with young fruits received a spray of preparation. Spraying was carried out until complete wetting of the upper trusses with young fruits, similar for all treatments. The experimental design was completely randomised and included 3 replicates with 10 plants. The experiment was arranged in 3 adjacent rows. Each replicate of each treatment was separated from another treatment by 5 plants that were not sprayed.

### 2.3. Sampling and Tissue Analysis

To assess the effect of treatments on the Ca concentration in the fruit, samples of two sizes of tomato fruit were collected from two consecutive bunches: the smallest fruits, on average 3 cm in diameter, and the largest from the next bunch, on average 6 cm in diameter. Samples were taken once a month from 21 October 2020 to 24 March 2021, 15–20 days after treatment.

The nutrient status of tomato plants in the greenhouse was diagnosed by analysing the leaves of the untreated control. Samples for chemical analysis of 12 essential nutrients (N, P, K, Ca, Mg, S, Fe, Mn, Zn, Cu, Mo, and B) were collected from fully developed youngest tomato leaves (under first flowering truss) and the older still vital leaves located under the fifth truss, two times per month from September 2020 to April 2021.

Air-dried tomato fruit and leaf samples were ground, dry ashed in concentrated HNO_3_ vapours, and the ash was dissolved in a 3% HCl solution. For N and S detection in tomato leaves, wet digestion in H_2_SO_4_ or HNO_3_ was used, respectively. Ca, K, and Mg content in the dry matter of tomato fruits was determined by microwave plasma atomic emission spectrometer (MP-AES) 4210 Agilent Technologies [24]. The levels of K, Ca, Mg, Fe, Cu, Zn, and Mn in tomato leaves were estimated by MP–AES 4210 Agilent Technologies, the levels of P, Mo, N, and B were determined by colorimetry, and S by turbidimetry with a spectrophotometer JENWAY 6300 as described previously [25]. The results were expressed as mass % and mg kg^−1^ on a dry matter basis for macronutrients and micronutrients, respectively.

### 2.4. Measurement of Yield Parameters

For all treatments, the total and marketable/non-marketable yield of fruits in grams per plant was recorded on a regular weekly basis during the 28-week harvest period from late October 2020 to the last commercial harvest at beginning of May 2021. The proportion of BER-affected fruit, which was the main cause of non-marketable tomato yield, was determined as the percentage of the total fruit yield per week.

### 2.5. Statistical Analysis

Mineral nutrition and yield data were analysed with descriptive statistics. A one-way ANOVA with post hoc Tukey HSD test and a Kruskal Wallis test with post hoc Wilcoxon test were conducted to determine whether there were any statistically significant differences between the Ca treatments’ effect on yield/nutrient levels and fruit size, respectively. Student’s *t*-test was performed to check the significance of the differences in the leaf nutrient concentrations between younger and older tomato leaves. The correlation analysis was determined using Pearson’s correlation analysis.

## 3. Results 

### 3.1. Nutrient Status

Leaf nutrient analyses were performed to diagnose possible imbalances that may affect plant vitality and, indirectly, Ca uptake. The data obtained showed that the content of most nutrients in tomato leaves (Table 1 and Table 2) corresponded to the standard range reported for tomato leaves [26,27,28]. The only deviations from the recommended were high S and low Zn content in both young and older tomato leaves. Although antagonistic effects are possible, our study did not reveal significant negative correlations between S and other nutrients in young and older tomato leaves. On the contrary, positive correlations were found for S content in young tomato leaves with N, K, Ca, and P (0.567 > *r* < 0.677, *p* = 0.05), for older leaves with P, K, Fe, and Cu (0.666 > *r* < 0.747, *p* = 0.05). Moreover, neither Ca deficiencies nor excessive K and Mg, nor a significant negative correlation between Ca-K and Ca–Mg in ‘Beorange’ leaves were found (Table 3 and Table 4). The obtained results indicated substantial differences in chemical composition between younger and older leaves. Significantly higher contents of Ca, S, Fe, Mn, Mo, and B in the oldest tomato leaves confirmed their low mobility and reuse in the plant. Therefore, in the oldest leaves the concentration of nutrients can often exceed the optimum values reported in the literature.

Thus, according to the leaf analyses, tomato plants were generally well supplied with nutrients and the mineral nutrition conditions could be considered suitable for conducting experiments with sprays of different Ca-containing or Ca uptake-promoting preparations to determine their effectiveness in increasing the Ca content in tomato fruits.

### 3.2. Ca Content in Tomato Fruits

**Figure 2:**
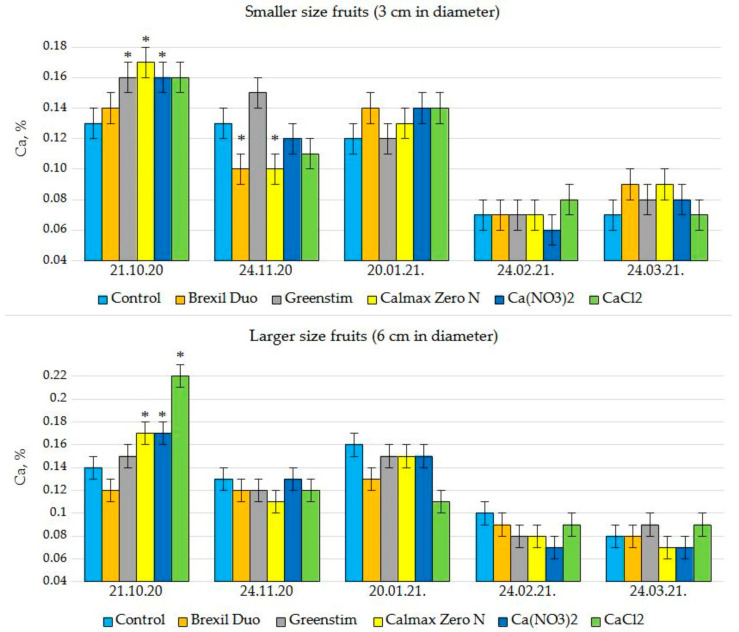
Effect of spraying of Ca-containing/Ca uptake-promoting preparations on Ca content (mass %, dry matter) in different size ‘Beorange’ tomato fruits at the early stages of fruit development (*n* = 3). Asterisk (*) indicates a statistically significant difference from the respective control at each sampling time (Kruskal Wallis test, *p* < 0.05).

In general, the results showed that the application of studied preparations did not significantly increase either the content of Ca in the young fruits at individual sampling times or the mean level of Ca throughout the growth cycle (Figure 2). The only exception was the first sampling on October 21. At this time, all sprays, except Brexil Duo for both size fruits and Greenstim for larger fruits, significantly increased the Ca content in fruits of the ‘Beorange’ variety. The first fruit sampling coincided with the beginning of the fruit harvest when the plants were relatively young; they had grown in the greenhouse for about two months. In addition, from the second half of October, solar radiation significantly decreased and artificial lighting increased in the greenhouse. The positive effect was no longer confirmed during the experiment as the plants aged towards the end of the growth cycle. During the autumn–spring growing cycle, a decrease in Ca content was found in tomato fruits for all treatments. Thus, the Ca content of both sizes of young fruits was only 0.08% on average at the last sampling times in late February and March.

### 3.3. Yield and BER Incidence

**Figure 3:**
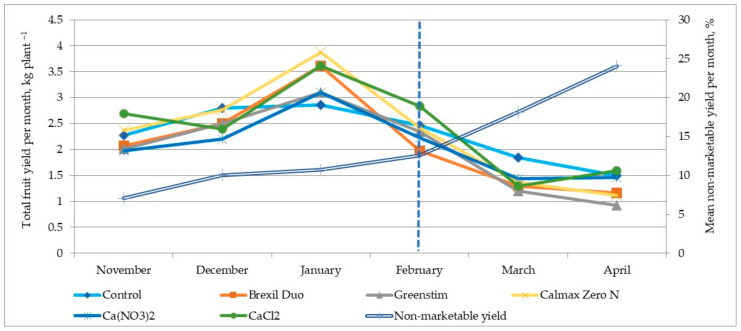
Total yield (kg plant^−1^) and mean non-marketable ‘Beorange’ tomato yield (% of total) per month in a greenhouse experiment with a spraying of different Ca-containing/Ca uptake-promoting preparations in the 2020/2021 season. The dashed vertical line indicates the end of February 2021, and a decrease in fruit Ca concentration.

Overall, the mean total fruit yield over the 28-week harvest period, as well as the pattern of total and non-marketable yield per month, were similar for all treatments and were not significantly affected (*p* < 0.05) by the Ca application to the fruits (Table 5, Figure 3).

Contrary to what was intended, the treatment of fruits with a solution of Brexil Duo, Greenstim, and Ca(NO_3_)_2_ resulted in approximately 10% lower yields compared to other treatments and untreated control, respectively. Although these differences were not statistically significant, they nevertheless indicated that the use of these preparations was irrelevant.

The percentage of non-marketable tomato yield was entirely determined by BER defects in the fruit, as no other fruit defects were detected. As a result of the spraying, no reduction in the proportion of BER-affected fruit was found in ‘Beorange’. During the experiment, the increase in the percentage of fruit affected by BER was not significantly different (*p* < 0.05) between all treatments and the control. The proportion of non-marketable yield increased at the end of the crop cycle (Figure 3) when the Ca content in the fruits decreased. Overall, the mean non-marketable yield for all treatments during the season averaged 15.11 ± 1.38% of the total yield.

Thus, from the end of February 2021 onwards, widespread BER incidence was observed, leading to an increase in the percentage of non-standard yield of up to 15% and even 25% at the end of the growing cycle. This coincided with a decrease in the Ca content of the fruit for all treatments (Figure 3).

Along with the Ca content in the fruits, the K and Mg content was also determined at all sampling times of tomato fruits. This was conducted to verify the possible influence of the Ca-containing/Ca uptake-promoting preparations on the potential Ca–Mg–K imbalance which could contribute to the development of BER. In this regard, our study did not reveal significant differences in fruit K and Mg content between fruits of both sizes or treatments, or sampling times. The average concentrations of K, Mg, and Ca in ‘Beorange’ fruits in two periods of the production cycle (October–January 2020 and February–March 2021) are given in Table 6. Therefore, the significantly higher mean fruit (K + Mg)/Ca ratios found in the second part of the growing season (sampling times: 24.02.21 and 24.03.21) were undeniably the result of lower Ca concentrations (Table 7).

## 4. Discussion

Optimal nutrient status is essential for the healthy growth of tomatoes in hydroponic systems and is one of the main factors determining the yield and its quality. Overall, leaf diagnostics revealed an adequate tomato supply with nutrients, thus indicating a high degree of precision in fertiliser management. According to different studies [23,29,30,31], competitive interaction between Ca, Mg, and K is a common phenomenon in tomato cultivation leading to the risk of physiological Ca disorders. In this regard, neither deficiencies nor imbalances in the supply of these nutrients were found. Indeed, even the lowest Ca concentrations in fully developed youngest tomato leaves corresponded to the reported 1.6% and above required for optimal fruit yield with minimal BER [32]. Moreover, the composition of the nutrient solution generally corresponded to the optimal Ca/Mg concentration for tomato cultivation, which was estimated at 300/50–80 mg L^−1^ [33] for the autumn greenhouse tomato crop. Other factors affecting the development of BER in tomatoes are closely related to the microclimate in the greenhouse [1,3,11]. As no significant deviations from the optimum air temperature, radiation level, and relative humidity recommended for tomato cultivation [23] were found, environmental conditions are unlikely to contribute to Ca-related disorders. Thus, generally suitable background conditions were provided for testing the effectiveness of Ca-containing/Ca uptake-promoting preparations.

For BER-sensitive varieties, tissue demand for Ca could exceed the supply of Ca, especially during the maximum growth rate and cell expansion period reported to occur approximately 10 to 20 days after anthesis [16,34]. In this regard, Ca sprays for decreasing BER were mainly found effective at the early stages of fruit development, from flowering to three weeks after anthesis [16]. With this consideration, in our study, all treatments were applied to young fruits immediately after the fruit initiations, before the onset of BER symptoms. Unfortunately, spraying fruits after fruit set with Ca-containing or Ca-stimulating preparations included in the study (Brexil Duo, Greenstim, Calmax Zero N, Ca(NO_3_)_2_, CaCl_2_) was not effective in increasing fruit Ca content. If, in the period of October–January, the fruit Ca content in most cases was in the range of 0.12–0.15%, then in February–March, it was only 0.07–0.09% (Figure 2). Although no specific critical Ca level directly related to the formation of BER has been identified, the studies indicated that the likelihood of BER is significantly increased if the Ca concentration in the tomato fruit (dry matter) falls below 0.08%, whereas disorders are rare if the Ca level in the fruit exceeds 0.12–0.20% [1,23]. The results obtained in our study also confirm the above concentrations and support the idea that Ca is indeed an important factor involved in the development of BER. However, imbalances of other nutrients in the fruits may also be involved [33]. Previous studies have shown that the incidence of BER may be related to a high fruit (K + Mg)/Ca ratio [5,35]. This is usually associated with increased fruit K and Mg content in BER-susceptible cultivars. The results of our study convincingly proved that a significantly higher fruit (K + Mg)/Ca ratio in the second half of the cropping cycle was not related to high K and Mg but to a decrease in Ca content. Moreover, there were no significant differences between the treatments. An increased (K + Mg)/Ca ratio due to decreased Ca rather than increased K and Mg concentrations was also reported for the BER-sensitive tomato cultivar ‘Reiyoh’ [35].

Unfortunately, since fruit spraying with Ca-containing or Ca-stimulating preparations was not effective in increasing Ca content, it was also not effective in promoting tomato fruit yield and preventing BER. Even the use of the most recommended and tested measure—sprays of CaCl_2_—did not show any effect on the Ca content of the fruit and the total yield. These findings were inconsistent with several previous studies [2,16,18,20,36] showing that foliar application of calcium fertilisers had positive effects on fruit production and quality.

The preparations used in the study contained Ca in four different forms of chemical compounds—chloride, nitrate, lignin sulfonate (Brexil Duo), and suspended oxide (Calmax Zero N). Despite the different compositions, none of the applied fertilisers were effective in increasing fruit Ca concentration. An increase in Ca content in fruits was detected only in the first sampling for CaCl_2_, Ca(NO_3_)_2_ and Calmax Zero N sprays. This is somewhat surprising in the case of Brexil Duo since the high complexing ability of nutrients with lignin sulphonate, natural selectivity, and facility to penetrate plant tissues are specified by the manufacturer as a feature of the fertilisers in the Brexil line [37]. It should be noted that this preparation also contains Mg and micronutrients (B, Cu, Mn, Zn) in small amounts. The fact that a nutrient complex intended to be supplied by foliar fertiliser could result in low absorption of a single nutrient is probably due to element antagonism and cation competition. This could be especially true for Ca and Mg. Because Ca and Mg have similar chemical properties and are absorbed through the same processes [7], these nutrients can inhibit each other. This could be evidenced by the ineffectiveness of the first sprayings of Brexil Duo, as the only Mg-containing Ca preparation, at a time when the other preparations nevertheless gave a small increase in Ca content in the fruits. In addition, treatment with a solution of Brexil Duo resulted in approximately 10% lower tomato yields compared to the control. In the case of the application of another preparation which contained small amounts of micronutrients, Calmax Zero N, neither positive nor negative effects were found on the Ca content and yield of tomato fruits. This suggests that a small dose of micronutrients in the composition of foliar Ca fertiliser could be beneficial in case of their deficiency but does not promote or inhibit Ca uptake by the plant. Therefore, to increase the content of Ca in fruits, it would not be recommended to use foliar fertilisers that contain Mg along with Ca.

Foliar fertilisation with Ca nitrate can lead to an increase not only in Ca but also in N concentration in tomato fruit, thus causing faster growth and a certain dilution of Ca levels in tomato fruit tissue. An increase in the fruit N and a decrease in Ca content as the result of foliar Ca nitrate fertilization was also previously reported for tomatoes [38]. The ineffectiveness of Ca nitrate application found in our study was probably related to this effect.

Contrary to expectations, no positive effects were found for the application of Greenstim, the only preparation that did not contain Ca. Glycine betaine can improve plant tolerance to salt or drought stress and nutrient uptake [39,40,41]. Precise control of greenhouse conditions significantly reduces abiotic stress (especially salt and water stress) compared to the natural environment (in the open field), where conditions are more variable, thus potentially reducing the effectiveness of this preparation. 

Usually, inefficiencies of additional Ca supply are associated with non-compliance with the technology; for example, spraying on the entire plant, only leaves, and irregular treatment [6]. In the case of our study, all preconditions for its effectiveness were generally met; Ca preparations were sprayed directly to the young tomato fruit immediately after the fruit set, before the onset of BER symptoms, and regularly throughout the growth cycle. It is possible that Ca uptake and BER induction could have been affected by many other factors, including the low solar radiation component in the autumn–winter period, the high pH level in the rock-wool substrate, and others. However, the results of the study showed that in the climatic conditions typical of Latvia and other surrounding countries, as well as following current recommendations for the hydroponic cultivation of tomatoes in rockwool, spraying of Ca preparations was not effective for the variety ‘Beorange’.

In general, an understanding of the environmental factors that affect both fruit growth rate and Ca transport to the fruit can improve cultivation practices to limit BER formation. To a large extent, all conditions and agronomic techniques recommended to reduce the formation of BER [23,26,27] were applied in Getlini ECO tomato greenhouses. Although studies have found that defoliation, by reducing leaf–fruit competition, is effective in reducing the incidence of BER in susceptible large-fruited cultivars such as ‘Momotaro fight’ [42,43], it did not produce the expected effect in ‘Beorange’.

Several studies have shown that the occurrence of Ca deficiency disorders in plants is closely related to phytohormones such as gibberellins (GA) and abscisic acid (ABA), especially in the early stages of fruit development [1,44,45]. They have the opposite effect on Ca uptake: GA can reduce Ca concentration, increasing stress sensitivity and BER occurrence, while ABA increases Ca translocation into the fruit, which prevents BER occurrence [8]. Therefore, foliar sprays with ABA alone or in combination with GA biosynthesis inhibitors are being investigated as an alternative treatment to increase Ca uptake in fruits [45,46]. This approach should be tested more widely as a possible technique to reduce fruit defects in BER-susceptible tomato cultivars.

## 5. Conclusions

The research results revealed that spraying the fruits after fruit set with the Ca-containing or Ca-stimulating preparations included in the study (Brexil Duo, Greenstim, Calmax Zero N, Ca(NO_3_)_2_, CaCl_2_) was not effective in increasing Ca content to prevent BER and did not contribute to the tomato fruit yield. As the supply of additional Ca is labour intensive and makes tomato production more expensive, it is not recommended as a BER solution for ‘Beorange’ in hydroponics on a rockwool substrate in the autumn–spring cycle. As good agricultural practices were followed in the greenhouse to manage BER, we concluded that a non-marketable yield of around 15% should be expected for the large-fruit variety ‘Beorange’ when grown in the autumn–spring growing cycle under artificial light, possibly due to the impact of abiotic stresses and genetically determined susceptibility. In general, the selection of tomato varieties for greenhouse cultivation with the desired fruit size, taste, and colour, while insensitive to BER, is very important. This is particularly true for tomato production in Latvia, considering that the geographical location in the hemiboreal climate zone makes growing vegetables expensive in the autumn–winter season due to lighting and heating costs.

## Figures and Tables

**Table 1:** Macronutrient concentration (mass %, dry matter) in young and older ‘Beorange’ tomato leaves during the crop cycle from September 2020 to April 2021. Nutrient sufficiency ranges for the youngest fully developed tomato leaves were indicated according to the established reference values [26,27,28].

Nutrient	Young Leaves (*n* = 14)		Older Leaves (*n* = 14)		Sufficiency Ranges in Tomato Leaves
	Range	Mean ± SE	Range	Mean ± SE	
N	3.50–5.15	4.01 ± 0.15b ^1^	3.20–4.00	3.44 ± 0.07a	3.50–6.00
P	0.46–0.66	0.52 ± 0.02a	0.57–0.89	0.76 ± 0.04b	0.30–1.00
K	2.69–6.50	4.25 ± 0.29a	3.07–7.20	4.91 ± 0.39a	3.50–6.00
Ca	1.55–5.59	2.87 ± 0.32a	4.95–7.30	6.13 ± 0.17b	1.20–4.00
Mg	0.27–0.55	0.37 ± 0.02a	0.25–0.58	0.41 ± 0.03a	0.30–1.00
S	0.70–2.08	1.28 ± 0.10a	1.88–3.63	2.67 ± 0.18b	0.40–1.00

^1^ For each nutrient, means within the row followed by the different letters are statistically different at the level of *p* < 0.05 (*t*–test, a < b).

**Table 2:** Micronutrient concentration (mg kg^−1^, dry matter) in young and older ‘Beorange’ tomato leaves during the crop cycle from September 2020 to April 2021. Nutrient sufficiency ranges for the youngest fully developed tomato leaves were indicated according to the established reference values [26,27,28].

Nutrient	Young Leaves (*n* = 14)		Old Leaves (*n* = 14)		Sufficiency Ranges in Tomato Leaves
	Range	Mean ± SE	Range	Mean ± SE	
Fe	133–323	209.38 ± 16.15a ^1^	180–549	326.36 ± 38.05b	100–300
Mn	68–132	93.77 ± 5.22a	127–300	188.45 ± 14.05b	50–200
Zn	14.0–31.5	19.79 ± 1.26a	9.5–35.0	19.18 ± 2.15a	25–80
Cu	10.5–22.0	16.43 ± 1.04b	6.5–21.5	11.30 ± 1.38a	6.0–25.0
Mo	1.50–5.00	3.40 ± 0.23a	2.88–8.00	4.83 ± 0.47b	1.0–5.0
B	24–54	32.62 ± 2.51a	38–84	60.73 ± 4.46b	25–75

^1^ For each nutrient, means within the row followed by the different letters are statistically different at the level of *p* < 0.05 (*t*–test, a < b).

**Table 3:** Pearson’s correlation matrix between nutrient concentrations in young tomato leaves during the crop cycle from September 2020 to April 2021.

	N	P	K	Ca	Mg	S	Fe	Mn	Zn	Cu	Mo
P	0.835 *	1.000									
K	0.577 *	0.627 *	1.000								
Ca	0.742 *	0.459	0.522 *	1.000							
Mg	0.808 *	0.486	0.443	0.798 *	1.000						
S	0.567 *	0.648 *	0.616 *	0.656 *	0.423	1.000					
Fe	−0.434	−0.161	0.282	−0.430	−0.455	0.059	1.000				
Mn	−0.522	−0.486	−0.257	−0.420	−0.536 *	−0.271	0.420	1.000			
Zn	−0.106	−0.056	−0.046	−0.142	0.117	−0.167	0.143	0.190	1.000		
Cu	0.237	0.345	0.193	0.184	0.347	0.190	−0.090	−0.293	0.636 *	1.000	
Mo	0.501 *	0.644 *	0.247	0.252	0.214	0.677 *	0.072	−0.278	0.084	0.186	1.000
B	0.352	0.442	0.475	0.334	0.198	0.366	0.279	−0.115	−0.246	0.130	0.095

Asterisk (*) indicates significance at *p* < 0.05, *r* > 0.497, *n* = 14.

**Table 4:** Pearson’s correlation matrix between nutrient concentrations in older tomato leaves during the crop cycle from September 2020 to April 2021.

	N	P	K	Ca	Mg	S	Fe	Mn	Zn	Cu	Mo
P	−0.402	1.000									
K	−0.048	0.772 *	1.000								
Ca	0.186	0.284	0.547	1.000							
Mg	0.606 *	−0.596 *	−0.255	−0.134	1.000						
S	0.028	0.719 *	0.747 *	0.060	−0.254	1.000					
Fe	−0.165	0.790 *	0.862 *	0.275	−0.064	0.666 *	1.000				
Mn	0.301	0.334	0.555 *	0.289	0.172	0.367	0.535 *	1.000			
Zn	0.290	0.086	0.374	0.176	0.508 *	0.399	0.354	0.407	1.000		
Cu	0.372	0.374	0.600 *	0.227	0.367	0.699 *	0.638 *	0.583 *	0.735 *	1.000	
Mo	−0.355	0.451	0.324	−0.058	−0.455	0.259	0.264	0.045	0.131	−0.064	1.000
B	−0.180	0.763 *	0.512	0.334	−0.616	0.531	0.424	0.273	−0.027	0.342	0.474

Asterisk (*) indicates significance at *p* < 0.05, *r* > 0.497, *n* = 14.

**Table 5:** Tomato yield of ‘Beorange’ variety obtained in the greenhouse experiment with sprays of different Ca-containing/Ca uptake-promoting preparations during the season 2020/2021 (cropping period of 28 weeks).

Treatment	Mean Total Yield, kg Plant^−1^	Compared to the Control, %
Control	15.31 ± 1.07	100
Brexil Duo 0.2%	13.65 ± 1.09	89
Greenstim 0.2%	13.62 ± 1.12	89
Calmax Zero N 0.5%	15.16 ± 1.39	99
Ca(NO_3_)_2_ 0.5%	13.74 ± 1.04	90
CaCl_2_ 0.5%	15.83 ± 1.29	103

There were no significant differences between treatments (ANOVA, *p* < 0.05).

**Table 6:** Mean K, Mg, and Ca concentration (mass %, dry matter) in ‘Beorange’ tomato fruits in two periods of the production cycle: October–January 2020 (sampling times: 21.10.20, 24.11.20, 20.01.21) and February–March 2021 (sampling times: 24.02.21, 24.03.21).

Nutrient	Smaller Size Fruits (3 cm in Diameter)		Larger Size Fruits (6 cm in Diameter)	
	October–January	February–March	October–January	February–March
K	4.10 ± 0.18a ^1^	4.33 ± 0.10a	4.01 ± 0.19a	4.39 ± 0.14a
Mg	0.20 ± 0.01a	0.19 ± 0.01a	0.17 ± 0.01a	0.17 ± 0.01a
Ca	0.13 ± 0.005b	0.08 ± 0.003a	0.14 ± 0.006b	0.08 ± 0.003a

^1^ For each nutrient, means within the row followed by the different letters are statistically different at the level of *p* < 0.05 (*t*–test, a < b).

**Table 7:** The effect of spraying different Ca-containing/Ca uptake-promoting preparations on the mean (K + Mg)/Ca ratio of ‘Beorange’ tomato fruits in two periods of the production cycle: October–January 2020 (sampling times: 21.10.20, 24.11.20, 20.01.21) and February–March 2021 (sampling times: 24.02.21, 24.03.21).

Treatment	Smaller Size Fruits (3 cm in Diameter)		Larger Size Fruits (6 cm in Diameter)	
	October–January	February–March	October–January	February–March
Control	34.19 ± 4.46a ^1^ A ^2^	71.64 ± 1.79abB	29.03 ± 2.97aA	54.90 ± 1.10aB
Brexil Duo, 0.2%	33.89 ± 0.97aA	56.82 ± 9.04aB	32.30 ± 4.01aA	52.83 ± 3.95aB
Greenstim, 0.2%	30.64 ± 5.36aA	64.68 ± 6.18aB	30.32 ± 2.62aA	56.85 ± 5.40aB
Calmax Zero N, 0.5%	32.08 ± 3.13aA	54.36 ± 9.36aB	29.07 ± 0.89aA	53.85 ± 6.72aB
Ca(NO_3_)_2_, 0.5%	30.60 ± 2.25aA	60.65 ± 8.02aB	27.36 ± 1.56aA	65.50 ± 2.36bB
CaCl_2_, 0.5%	32.18 ± 2.04aA	58.93 ± 2.93aB	30.74 ± 6.46aA	49.78 ± 4.33aB

Values with different letters differ significantly (ANOVA, *p* < 0.05). ^1^ For columns, lowercase letters compare treatments for each period and fruit size (a < b). ^2^ For rows, uppercase letters compare periods for each fruit size and treatment (A < B).

## Data Availability

All data reported here are available from the authors upon request.
